# Haplotype-Based Genome-Wide Prediction Models Exploit Local Epistatic Interactions Among Markers

**DOI:** 10.1534/g3.117.300548

**Published:** 2018-03-16

**Authors:** Yong Jiang, Renate H. Schmidt, Jochen C. Reif

**Affiliations:** Department of Breeding Research, Leibniz Institute of Plant Genetics and Crop Plant Research (IPK) Gatersleben, 06466 Stadt Seeland, Germany

**Keywords:** haplotype, epistasis, local epistatic effect, genome-wide prediction, GenPred, Shared Data Resources, Genomic Selection

## Abstract

Genome-wide prediction approaches represent versatile tools for the analysis and prediction of complex traits. Mostly they rely on marker-based information, but scenarios have been reported in which models capitalizing on closely-linked markers that were combined into haplotypes outperformed marker-based models. Detailed comparisons were undertaken to reveal under which circumstances haplotype-based genome-wide prediction models are superior to marker-based models. Specifically, it was of interest to analyze whether and how haplotype-based models may take local epistatic effects between markers into account. Assuming that populations consisted of fully homozygous individuals, a marker-based model in which local epistatic effects inside haplotype blocks were exploited (LEGBLUP) was linearly transformable into a haplotype-based model (HGBLUP). This theoretical derivation formally revealed that haplotype-based genome-wide prediction models capitalize on local epistatic effects among markers. Simulation studies corroborated this finding. Due to its computational efficiency the HGBLUP model promises to be an interesting tool for studies in which ultra-high-density SNP data sets are studied. Applying the HGBLUP model to empirical data sets revealed higher prediction accuracies than for marker-based models for both traits studied using a mouse panel. In contrast, only a small subset of the traits analyzed in crop populations showed such a benefit. Cases in which higher prediction accuracies are observed for HGBLUP than for marker-based models are expected to be of immediate relevance for breeders, due to the tight linkage a beneficial haplotype will be preserved for many generations. In this respect the inheritance of local epistatic effects very much resembles the one of additive effects.

Genome-wide regression is a powerful tool to analyze and predict quantitative traits which are regulated by many genes (Meuwissen *et al.* 2001). Various genome-wide prediction approaches have been explored and applied for human (Yang *et al.* 2010, de los Campos *et al.* 2010, 2013b), animal (Hayes *et al.* 2013, de los Campos *et al.* 2013a), and plant populations (Crossa *et al.* 2014, Heslot *et al.* 2015, Hickey *et al.* 2017). In most genome-wide prediction models, effects of molecular markers such as single nucleotide polymorphisms (SNPs) were used as explanatory variables (Cuyabano *et al.* 2015a). Alternatively, molecular markers can be combined into haplotypes, which are then used to implement genome-wide prediction models (Calus *et al.* 2008). Haplotype-based prediction approaches are favored if alleles at quantitative trait loci (QTL) were more closely linked to haplotype alleles than individual SNPs (Zondervan and Cardon 2004). Moreover, it is hypothesized that haplotypes can capture epistatic interactions between SNPs (Clark 2004, Zhang *et al.* 2014). Therefore, haplotype-based approaches potentially boost prediction accuracies (Cuyabano *et al.* 2014, 2015a, b).

The potential to exploit local epistatic effects among markers in haplotype-based prediction is interesting with respect to two points. First, epistasis has been recognized as a biologically influential component contributing to the genetic architecture of quantitative traits (Carlborg and Haley 2004, Mackay 2014, Jiang *et al.* 2017). The role of epistasis in genome-wide prediction has been extensively studied, but mostly in terms of marker-based approaches. Several marker-based models either implicitly or explicitly including epistatic effects in addition to main effects were developed (Xu 2007, Gianola and van Kaam 2008, Wittenburg *et al.* 2011, Jiang and Reif 2015, Vitezica *et al.* 2017). Taking epistasis into account can increase prediction accuracies (Wang *et al.* 2012, Muñoz *et al.* 2014, He *et al.* 2016). Second, decomposing epistasis into global and local effects is pivotal for evaluating the long-term impact of epistasis in plant and animal breeding, as there is a reduced chance that local epistatic effects will disappear after generations of recombination (Akdemir and Jannink 2015). First attempts in exploiting additive and local epistatic effects for genome-wide prediction were carried out with marker-based models resulting in good predictive performance and useful explanatory information (Akdemir and Jannink 2015, Akdemir *et al.* 2017, He *et al.* 2017). Nevertheless, it has not been clarified why and how the haplotype-based approaches take local epistasis into account at the level of statistical models.

The aims of this study were 1) to provide a formal theoretical explanation how haplotype-based genome-wide prediction models intrinsically exploit local epistatic effects among markers, 2) to investigate with simulation studies under which circumstances haplotype-based models perform better than marker-based models, and 3) to explore the potential of haplotype-based genome-wide prediction models using three published empirical data sets.

## THEORY

This section was organized as follows: First we introduced two genome-wide prediction models. Haplotype effects were used as explanatory variables in the haplotype-based genomic best linear unbiased prediction (HGBLUP) model, while additive and local epistatic effects among markers were utilized as predictors in the locally extended genomic best linear unbiased prediction (LEGBLUP) model. Then we proved that the haplotype-based model HGBLUP exploits local epistatic effects among markers by establishing a link between HGBLUP and LEGBLUP for the case in which all loci are homozygous. At the end of section, two examples were given to illustrate the theoretical results.

Throughout the section, we made following conventions: Let n be the number of genotypes, p be the number of markers. In this study we only considered bi-allelic markers. Suppose that the whole genome is divided into non-overlapping haplotype blocks; local epistasis is defined as interaction effects among two or more markers within a defined haplotype block. Let w be the number of blocks. For 1≤k≤w, let $p_{k}$ be the number of markers in the k-th block. Let $s_{k}$ be the number of different haplotype alleles in the k-th block. Linkage phases were assumed to be known. Vectors (matrices) are always denoted by lower (upper) case Latin or Greek letters in bold font.

### The HGBLUP model

This model has been used in previous studies (*e.g.*, Cuyabano *et al.* 2014, 2015a) and here we called it HGBLUP. Independent from the definition of haplotype blocks, the HGBLUP model can be described as follows:

$$y=1_{n}\mu +\overset{w}{\underset{k=1}{\sum }}X_{k}h_{k}+e,$$ [1]

where y is the n-dimensional vector of phenotypic records, $1_{n}$ is an n-dimensional vector of one’s, μ is a common intercept term, $h_{k}$ is the $s_{k}$-dimensional vector of haplotype effects in the k-th haplotype block, $X_{k}$ is the corresponding $n\times s_{k}$ design matrix of the k-th block, the (i,j)-entry of $X_{k}$ is the number of the j-th haplotype allele in the i-th genotype (hence, it is 0, 1 or 2), and e is the residual term. In the model we assumed that μ is a fixed parameter, $h_{k}∼N(0,I_{s_{k}}\sigma_{h}^{2})$ for any k, and $e∼N(0,I_{n}\sigma_{e}^{2})$. We assumed no covariance structure among these variables.

The formulation of this model is similar to ridge regression best linear unbiased prediction (RR-BLUP, Meuwissen *et al.* 2001) except that the marker effects were replaced by haplotype effects. Note that there are in total $1+\overset{w}{\underset{k=1}{\sum }}s_{k}$ unknown parameters in the model. This number can be even larger than the number of markers, which makes the computational load very high. However, the model can be implemented in an alternative way similar to the marker-based genomic best linear unbiased prediction model (GBLUP, VanRaden 2008):

$$y=1_{n}\mu +g+e,$$ [2]

where y, $1_{n}$, μ, and e are the same as in Equation 1; g is an n-dimensional vector of genotypic values. We assumed that μ is a fixed parameter, $e∼N(0,I_{n}\sigma_{e}^{2})$, and $g∼N(0,H\sigma_{g}^{2})$, where $H=\frac{1}{p}\overset{w}{\underset{k=1}{\sum }}X_{k}X_{k}\prime$. Setting $\sigma_{g}^{2}=p\sigma_{h}^{2}$, it becomes obvious that the two models are statistically equivalent, as the equivalence between GBLUP and RR-BLUP (Habier *et al.* 2007).

### The LEGBLUP model

This model is a local version of the extended GBLUP (EGBLUP) (Jiang and Reif 2015). EGBLUP exploits epistasis between any pair of markers while LEGBLUP only considers local epistasis inside each haplotype block. Assuming only digenic epistasis, the model can be described as follows:

$$y=1_{n}\mu +\mathrm{Ma}+\overset{w}{\underset{k=1}{\sum }}F_{k}aa_{k}+e,\;$$ [3]

where y, $1_{n}$, μ, and e are the same as in Equation 1, M is the n×p matrix of marker profiles, the (i,j)-entry of M is the number of a specific allele of the j-th marker carried by the i-th genotype (hence, it is 0, 1 or 2), a is the p-dimensional vector of marker additive effects, $F_{k}$ is the $n\times \frac{p_{k}(p_{k}-1)}{2}$ design matrix for additive-by-additive epistatic effects for markers in the k-th haplotype block, $aa_{k}$ is the $\frac{p_{k}(p_{k}-1)}{2}$-dimensional vector of epistatic effects in the k-th block. In the model we assumed that μ is a fixed parameter, $a∼N(0,I_{n}\sigma_{a}^{2})$, $aa_{k}∼N\left(0,I\sigma_{aa}^{2}\right)$ for any k, and $e∼N(0,I_{n}\sigma_{e}^{2})$. We assumed no covariance structure among these variables.

Note that there are 1+p+q unknown variables in the model with $q=\frac{1}{2}\overset{w}{\underset{k=1}{\sum }}p_{k}(p_{k}-1)$, and this number can be very large. Hence, the model can be implemented in an alternative way as:

$$y=1_{n}\mu +g_{1}+g_{2}+e,$$ [4]

where y, $1_{n}$, μ, and e are the same as in Equation 1, $g_{1}$ is an n-dimensional vector of additive genotypic values, $g_{2}$ is an n-dimensional vector of genetic values accounting for local epistasis. We assumed that μ is a fixed parameter, $e∼N(0,I_{n}\sigma_{e}^{2})$, $g_{1}∼N(0,G_{1}\sigma_{g_{1}}^{2})$ and $g_{2}∼N(0,G_{2}\sigma_{g_{2}}^{2})$, where $G_{1}=\frac{1}{p}\mathrm{MM}\prime$, $G_{2}=\frac{1}{2q}\overset{w}{\underset{k=1}{\sum }}[(M_{k}M_{k}\prime )\#(M_{k}M_{k}\prime )-\left(M_{k}\#M_{k}\right)\left(M_{k}\#M_{k}\right)\prime ]$, and # is the Hadamard product, *i.e.*, entry-wise product, of matrices. Setting $\sigma_{g_{1}}^{2}=p\sigma_{a}^{2}$ and $\sigma_{g_{2}}^{2}=q\sigma_{aa}^{2}$, it reveals that the two models are statistically equivalent. The reason is the same as the equivalence between EGBLUP and an extended RR-BLUP model including epistasis (Jiang and Reif 2015).

Note that in the above descriptions the LEGBLUP model only includes digenic local epistasis, *i.e.*, local epistatic effects between two markers. In fact, the LEGBLUP model can be generalized to include all possible higher-order epistatic interaction effects within each haplotype block, as the EGBLUP model. Briefly, we only need to extend Equation 4 to:

$$y=1_{n}\mu +g_{1}+g_{2}+\cdots +g_{r}+e,$$ [5]

where $g_{t}$ is the vector of genetic values accounting for (r-1)-th order epistasis, *i.e.*, epistatic interactions among r markers. The kinship matrix for $g_{t}$ can be derived using t-fold Hadamard product of $G_{1}$ (Jiang and Reif 2015). This model is denoted as full LEGBLUP.

### The link Between HGBLUP and LEGBLUP

We first concentrated on a single haplotype block, thus subscripts to differentiate blocks can be ignored. We assumed p markers and s haplotype alleles and then the HGBLUP model (Equation 1) reduces to:

$$y=1_{n}\mu +\mathrm{Xh}+e,$$ [6]

where h is an s-dimensional vector of haplotype effects and X is an n×s design matrix. The assumptions were that μ is a fixed parameter, $h∼N(0,I_{s}\sigma_{h}^{2})$, and $e∼N(0,I_{n}\sigma_{e}^{2})$.

For LEGBLUP, a unified expression of Equation 3 was needed to extend it to full LEGBLUP. We defined **α** to be a vector whose components are marker main effects together with epistatic effects up to the (p−1)-th order, *i.e.*, all possible epistatic effects among any number of markers in the block, not only digenic epistatic effects. Thus, the dimension of α is:

$$p+\left(\begin{array}{c} p\\ 2 \end{array}\right)+\cdots +\left(\begin{array}{c} p\\ p \end{array}\right)=2^{p}-1,$$

where $\left(\begin{array}{c} a\\ b \end{array}\right)=\frac{a(a-1)\cdots (a-b+1)}{b(b-1)\cdots 1}$ denote the Gaussian binomial coefficients. Let Z be the corresponding $n\times \left(2^{p}-1\right)$ design matrix. With these notations, the full LEGBLUP model can be simply written as:

$$y=1_{n}\mu +\mathrm{Z\alpha}+e.$$ [7]

The assumptions were that μ is a fixed parameter, α∼N(0,D), $e∼N(0,I_{n}\sigma_{e}^{2})$, and D is a diagonal matrix containing different unknown variance parameters for additive effects and different orders of epistatic effects.

#### Claim:

If all loci under consideration are homozygous, then there exists a $\left(2^{p}-1\right)\times \text{s}$ matrix V such that X=ZV.

The above claim was the key to bridge HGBLUP and LEGBLP. As its proof requires more techniques in linear algebra, we presented it as a separate subsection below.

Now we assumed all loci to be homozygous. Setting β= Vh, HGBLUP (Equation 6) can be expressed as:

$$y=1_{n}\mu +\mathrm{Z\beta}+e.\;$$ [8]

The newly defined vector β has the same design matrix as α in the LEGBLUP model (Equation 7). Thus, β includes marker effects as well as epistatic effects among markers. Accordingly, Equation 8 is the same as Equation 7 and hence HGBLUP has the same base equation as LEGBLUP.

Nevertheless, there is one important difference between the two models. In LEGBLUP, the covariance matrix for α is assumed to be a diagonal matrix D, hence, no covariance between different variables is assumed. But in HGBLUP, although the distribution of β is still multivariate normal, its covariance structure is:

$$\text{cov}\left(\beta \right)=V\text{cov}\left(h\right)V^{\prime }=\mathrm{VV}\prime \sigma_{h}^{2}.$$

In general, the matrix VV′ is semi-positive definite but not diagonal. Thus, HGBLUP implicitly assumes a non-trivial covariance structure.

Now it is straightforward to generalize the results to the case of a full model including all blocks, since no inter-block effects are modeled and the linear transformation X=ZV can be independently applied to each block.

### The proof of the claim

As the loci under consideration are homozygous, there are $\left(2^{p}-1\right)$ independent variables in the LEGBLUP model (Equation 7). For the HGBLUP model, there are at most $2^{p}$ different haplotype alleles, *i.e.*, $s\leq 2^{p}$. But note that if there are s haplotype alleles, the number of independent variables in the model is s−1 because of collinearity, similar to the biallelic case (*e.g.*, SNP markers) in which there is only one independent variable. Hence, we can assume $s\leq 2^{p}-1$. We shall consider two cases.

#### Case 1: All possible haplotype alleles occur:

We assumed that all possible haplotype alleles occur in the data, then $s=2^{p}-1$. We started from the HGBLUP model (Equation 6). Recall that for any 1≤i≤n and 1≤j≤s, the (i,j)-entry of X, denoted by $x_{ij}$, is the number of the j-th haplotype allele carried by the i-th individual. Since all marker loci are homozygous, $x_{ij}$ must be 0 or 2. As we assumed that all possible haplotype alleles occur in the data, for any j (1≤j≤s) there exists $i_{j}$ ($1\leq i_{j}\leq n$) such that $x_{i_{j}j}=2$ and $x_{i_{j}k}=0$ for all 1≤k≤s and k≠j. Combining the s rows $x_{i_{1}},\;x_{i_{2}},\cdots ,x_{i_{s}}$ of the design matrix X results in an s×s submatrix $\tilde{X}$. It is clear that $\tilde{X}$ is invertible because it can be transformed to $2I_{s}$ by row permutation. Correspondingly, we took the s rows $z_{i_{1}},\;z_{i_{2}},\cdots ,z_{i_{s}}$ of the design matrix Z in the LEGBLUP model (Equation 7). This also yielded an s×s submatrix $\tilde{Z}$. We observed that $\tilde{Z}$ is also invertible. The proof of this fact was presented separately at the end of this subsection. As $\tilde{Z}$ is invertible, we can define $V={\tilde{Z}}^{-1}\tilde{X}$ and hence $\tilde{X}=\tilde{Z}V$ with V being invertible.

We then claimed that X=ZV. In fact, for any $l\notin \{i_{1},i_{2},\cdots ,i_{s}\}$ and 1≤l≤n, the l-th row $x_{l}$ of X must coincide with $x_{i_{t}}$ for some 1≤t≤s because $x_{i_{1}},\;x_{i_{2}},\cdots ,x_{i_{s}}$ exhaust all the possibilities of row vectors for X. Correspondingly, the l-th row $z_{l}$ of Z must coincide with $z_{i_{t}}$. Since $\tilde{X}=\tilde{Z}V$ and $x_{l}$, $z_{l}$ are corresponding rows in $\tilde{X}$ and $\tilde{Z}$, $x_{l}=z_{l}V$. As it holds for any l, X=ZV.

#### Case 2: Not all possible haplotype alleles occur:

Now we assumed that not all possible haplotype alleles occur in the data ($s<2^{p}-1$). In contrast to the case that considers all haplotype alleles, the submatrix $\tilde{Z}$ in the LEGBLUP is not s×s but $s\times (2^{p}-1)$. So we need to adjust our arguments. In fact, $\tilde{Z}$ has full row rank: using the results in the previous case, $\tilde{Z}$ can be viewed as a submatrix of a full rank $\left(2^{p}-1)\times (2^{p}-1\right)$ matrix. Hence, there exists a right inverse W which is a $(2^{p}-1)\times s$ matrix such that $\tilde{Z}W=I_{s}$. Defining $V=W\tilde{X}$, we still obtain $\tilde{X}=\tilde{Z}V$, and hence X=ZV.

#### The proof of the fact that $\tilde{Z}$ is invertible:

Recall that *$\tilde{Z}$* is an s×s matrix, where $s=2^{p}-1$. The columns of $\tilde{Z}$ can be naturally indexed by the set

$$\text{Ψ}=\{(j_{1},j_{2},\cdots ,\;j_{t})|1\leq t\leq p,1\leq j_{1}<j_{2}<\cdots <j_{t}\leq p\}.$$

In fact we can denote the entries in $\tilde{Z}$ by *$\;{\tilde{z}}_{j_{1}j_{2}\cdots j_{t}}^{i}$*. When t=1, $\;{\tilde{z}}_{j_{1}}^{i}$is just the number of alleles of the $j_{1}$-th marker carried by the i-th genotype, which serves as the coefficient for the main additive effect of the $j_{1}$-th marker. When t≥2, $\;{\tilde{z}}_{j_{1}j_{2}\cdots j_{t}}^{i}=\;{\tilde{z}}_{j_{1}}^{i}\cdot {\tilde{z}}_{j_{2}}^{i}\cdots \;{\tilde{z}}_{j_{t}}^{i}$ is the coefficient of the epistatic effects among the markers $j_{1}$, $j_{2}$,… and$\;j_{t}$ for the i-th genotype. With the above notations, the column vectors of ***$\tilde{Z}$*** can be denoted by $\tilde{z}{\;}_{j_{1}j_{2}\cdots j_{t}}$.

The rows of *$\tilde{Z}$* can also be labeled by the set Ψ, which is trivial because *$\tilde{Z}$* has the same number of rows as columns. But we can introduce the following natural labeling: If a genotype is coded as 2 in the markers $j_{1}$, $j_{2}$,…,$\;j_{t}$ and 0 in the remaining ones, then we label the corresponding row as $\left(j_{1},j_{2},\cdots ,\;j_{t}\right)$. With these notations, the entries in *$\tilde{Z}$* can be written as $\;{\tilde{z}}_{j_{1}j_{2}\cdots j_{t}}^{i_{1}i_{2}\cdots i_{r}}$, where 1≤t,r≤p, $1\leq j_{1}<j_{2}<\cdots <j_{t}\leq p$, $1\leq i_{1}<i_{2}<\cdots <i_{r}\leq p$. By definition we have:

$$\;{\tilde{z}}_{j_{1}j_{2}\cdots j_{t}}^{i_{1}i_{2}\cdots i_{r}}=\{\begin{array}{c} 2^{t},\;\;\;\;\text{if}\;t\leq r\;\text{and}\;\left\{j_{1}j_{2}\cdots j_{t}\right\}\subseteq \{i_{1}i_{2}\cdots i_{r}\}\\ 0,\text{otherwise}. \end{array}$$ [9]

To show that $\tilde{Z}$ is invertible, it is sufficient to show that the column vectors ${\tilde{z}}_{j_{1}j_{2}\cdots j_{t}}$ (1≤t≤p, $1\leq j_{1}<j_{2}<\cdots <j_{t}\leq p$) span the space ${\mathbb{Q}}^{s}$, where ℚ denotes the set of rational numbers. The space ${\mathbb{Q}}^{s}$ has a natural basis $\{\varepsilon_{j_{1}j_{2}\cdots j_{t}}|1\leq t\leq p$, $1\leq j_{1}<j_{2}<\cdots <j_{t}\leq p\}$, where $\varepsilon_{j_{1}j_{2}\cdots j_{t}}$ is the vector whose $\left(j_{1},j_{2},\cdots ,\;j_{t}\right)$-entry is 1 and all other entries are zeros.

We first considered t=p. In this case we have only one vector ${\tilde{z}}_{12\cdots p}$, which is the coefficient of the epistatic effects among all p markers. From Equation 9 we know that the only non-zero entry in ${\tilde{z}}_{12\cdots p}$ is ${\tilde{z}}_{12\cdots p}^{12\cdots p}$ and it equals $2^{p}$. So $\varepsilon_{12\cdots p}=\frac{1}{2^{p}}{\tilde{z}}_{12\cdots p}$.

Next we considered the case t=p−1. In this case we have p vectors ${\tilde{z}}_{1\cdots \hat{k}\cdots p}$, where $\hat{k}$ denotes that k is absent in the sequence 1,2,…,p. Again using Equation 9, we know that there are only two non-zero entries in ${\tilde{z}}_{1\cdots \hat{k}\cdots p}$, namely $\;{\tilde{z}}_{1\cdots \hat{k}\cdots p}^{1\cdots p}$ and $\;{\tilde{z}}_{1\cdots \hat{k}\cdots p}^{1\cdots \hat{k}\cdots p}$, both values are $2^{p-1}$. Hence, $\varepsilon_{1\cdots \hat{k}\cdots p}=\frac{1}{2^{p-1}}{\tilde{z}}_{1\cdots \hat{k}\cdots p}-\frac{1}{2^{p}}{\tilde{z}}_{12\cdots p}$.

Repeating the procedure for smaller t, we can see that all basis vectors $\varepsilon_{j_{1}j_{2}\cdots j_{t}}$ can be written as linear combinations of the vectors $\tilde{z}{\;}_{j_{1}j_{2}\cdots j_{t}}$, which completes the proof.

### The case of heterozygous loci

Recall Equation 6 for HGBLUP and Equation 7 for LEGBLUP. Different from the case in which homozygous loci are considered, now the elements $x_{ij}$ in the design matrix X can take the value 1, in addition to 0 and 2. More precisely, when the paternal and maternal haplotypes are different, the corresponding row vector of X will have two non-zero entries, both being 1. This essential difference makes it impossible to find a matrix V such that X=ZV holds in general. So there does not exist any linear transformation β=Vh such that the base equations of HGBLUP and LEGBLUP become the same. This result was proved by giving a counterexample (see Example 2 in the next subsection).

### Illustration of the theoretical results

In this section, two examples were provided illustrating the theoretical findings for homozygous (Example 1; Table 1) and heterozygous loci (Example 2; Table 2).

**Table 1:** Summary of SNP marker coding and haplotype alleles for the six individuals considered in Theory, Example 1

**Individual**	**SNP1**	**SNP2**	**Hap1**	**Hap2**
**1**	2	2	11	11
**2**	2	0	10	10
**3**	0	2	01	01
**4**	0	0	00	00
**5**	2	0	10	10
**6**	2	2	11	11

**Table 2:** Summary of SNP coding and haplotype alleles for the 6 individuals considered in Theory, Example 2

**Individual**	**SNP1**	**SNP2**	**Hap1**	**Hap2**
**1**	2	2	11	11
**2**	2	0	10	10
**3**	0	2	01	01
**4**	2	1	11	10
**5**	1	0	10	00
**6**	1	1	10	01

#### Example 1:

We considered six individuals and one haplotype block with two SNP markers (Table 1). As outlined above the two homozygous genotypes were coded as 0 and 2 resulting in four different haplotype alleles.

The vector of haplotype effects is $h=(h_{11},h_{10},h_{01},h_{00})\prime$ and the corresponding design matrix is $X=\left(\begin{array}{cccc} 2 & 0 & 0 & 0\\ 0 & 2 & 0 & 0\\ 0 & 0 & 2 & 0\\ 0 & 0 & 0 & 2\\ 0 & 2 & 0 & 0\\ 2 & 0 & 0 & 0 \end{array}\right)$. As the fourth column of X can be obtained by subtracting the sum of the other three columns in the vector (2,2,2,2,2,2)′, the last variable can be dropped resulting in $h=(h_{11},h_{10},h_{01})\prime$ and $X=\left(\begin{array}{ccc} 2 & 0 & 0\\ 0 & 2 & 0\\ 0 & 0 & 2\\ 0 & 0 & 0\\ 0 & 2 & 0\\ 2 & 0 & 0 \end{array}\right)$. Then the HGBLUP model has the following form:

$$y=1_{n}\mu +\mathrm{Xh}+e=\left(\begin{array}{c} 1\\ 1\\ 1\\ 1\\ 1\\ 1 \end{array}\right)\mu +\left(\begin{array}{ccc} 2 & 0 & 0\\ 0 & 2 & 0\\ 0 & 0 & 2\\ 0 & 0 & 0\\ 0 & 2 & 0\\ 2 & 0 & 0 \end{array}\right)\left(\begin{array}{c} h_{11}\\ h_{10}\\ h_{01} \end{array}\right)+e,$$ [10]

with the assumptions $h∼N\left(0,I_{3}\sigma_{h}^{2}\right)$, $e∼N\left(0,I_{6}\sigma_{e}^{2}\right)$.

The vector of marker effects is $\alpha =(a_{1},a_{2},aa_{12})\prime$ with the design matrix $Z=\left(\begin{array}{ccc} 2 & 2 & 4\\ 2 & 0 & 0\\ 0 & 2 & 0\\ 0 & 0 & 0\\ 2 & 0 & 0\\ 2 & 2 & 4 \end{array}\right)$ with the third column of Z being the element-wise product of the first two columns; so the LEGBLUP model has the form:

$$y=1_{n}\mu +\mathrm{Z\alpha}+e=\left(\begin{array}{c} 1\\ 1\\ 1\\ 1\\ 1\\ 1 \end{array}\right)\mu +\left(\begin{array}{ccc} 2 & 2 & 4\\ 2 & 0 & 0\\ 0 & 2 & 0\\ 0 & 0 & 0\\ 2 & 0 & 0\\ 2 & 2 & 0 \end{array}\right)\left(\begin{array}{c} a_{1}\\ a_{2}\\ aa_{12} \end{array}\right)+e,\;$$ [11]

with the assumptions $\alpha =\left(\begin{array}{c} a_{1}\\ a_{2}\\ aa_{12} \end{array}\right)∼N(0,\left(\begin{array}{ccc} \sigma_{a}^{2} & 0 & 0\\ 0 & \sigma_{a}^{2} & 0\\ 0 & 0 & \sigma_{aa}^{2} \end{array}\right))$, $e∼N\left(0,I_{6}\sigma_{e}^{2}\right)$.

We took the first three rows in X and formed the submatrix $\tilde{X}$, as each of the first three individuals carries a different haplotype allele. So $\tilde{X}=2I_{3}$. Then we accordingly took the first three rows in Z to form the submatrix **$\tilde{Z}=\left(\begin{array}{ccc} 2 & 2 & 4\\ 2 & 0 & 0\\ 0 & 2 & 0 \end{array}\right)$** and defined $V={\tilde{Z}}^{-1}\tilde{X}=\left(\begin{array}{ccc} 0 & 1 & 0\\ 0 & 0 & 1\\ \frac{1}{2} & -\frac{1}{2} & -\frac{1}{2} \end{array}\right)$, thus, X=ZV. Assuming β=Vh resulted in Xh=ZVh=Zβ with

$$\text{cov}\left(\beta \right)=V\text{var}\left(h\right)V^{\prime }=\left(\begin{array}{ccc} 1 & 0 & -\frac{1}{2}\\ 0 & 1 & -\frac{1}{2}\\ -\frac{1}{2} & -\frac{1}{2} & \frac{3}{4} \end{array}\right)\sigma_{h}^{2}.$$

Hence, the HGBLUP model (Equation 10) is equivalent to

$$y=1_{n}\mu +\mathrm{Z\beta}+e,\;$$ [12]

with the assumptions $\beta =\left(\begin{array}{c} \beta_{1}\\ \beta_{2}\\ \beta_{3} \end{array}\right)∼N(0,\left(\begin{array}{ccc} \sigma_{h}^{2} & 0 & -\frac{1}{2}\sigma_{h}^{2}\\ 0 & \sigma_{h}^{2} & -\frac{1}{2}\sigma_{h}^{2}\\ -\frac{1}{2}\sigma_{h}^{2} & -\frac{1}{2}\sigma_{h}^{2} & \frac{3}{4}\sigma_{h}^{2} \end{array}\right))$, $e∼N\left(0,I_{6}\sigma_{e}^{2}\right)$.

Since in Equation 12 the parameters β have exactly the same design matrix as α in Equation 11, the base equations of HGBLUP and LEGBLUP are indeed the same. Setting $\sigma_{a}^{2}=\sigma_{h}^{2}$ and $\sigma_{aa}^{2}=\frac{3}{4}\sigma_{h}^{2}$, we can see that the only difference between the models is that in LEGBLUP (Equation 11) the covariance between additive and epistatic effects was zero while in HGBLUP (Equation 12) the covariance was $-\frac{1}{2}\sigma_{h}^{2}$.

#### Example 2:

We considered six genotypes and a haplotype block with two SNP markers (Table 2). In contrast to Example 1, we assumed presence of heterozygous loci. The vector of haplotype effects is $h=(h_{11},h_{10},h_{01},h_{00})\prime$ with the design matrix $X=\left(\begin{array}{cccc} 2 & 0 & 0 & 0\\ 0 & 2 & 0 & 0\\ 0 & 0 & 2 & 0\\ 1 & 1 & 0 & 0\\ 0 & 1 & 0 & 1\\ 0 & 1 & 1 & 0 \end{array}\right)$. As outlined in the first example, we can simply set $h=(h_{11},h_{10},h_{01})\prime$ owing to linear dependency and $X=\left(\begin{array}{ccc} 2 & 0 & 0\\ 0 & 2 & 0\\ 0 & 0 & 2\\ 1 & 1 & 0\\ 0 & 1 & 0\\ 0 & 1 & 1 \end{array}\right)$. The vector of marker effects is $\alpha =(a_{1},a_{2},aa_{12})\prime$ with the design matrix $Z=\left(\begin{array}{ccc} 2 & 2 & 4\\ 2 & 0 & 0\\ 0 & 2 & 0\\ 2 & 1 & 2\\ 1 & 0 & 0\\ 1 & 1 & 1 \end{array}\right)$.

In the following, we showed that there does not exist any matrix V such that X=ZV, *i.e.*, the HGBLUP and LEGBLUP model have the same base equations. For the proof, we assumed the contrary, that there exists a matrix V such that X=ZV. Then for any submatrix $\tilde{X}$ of X and the corresponding submatrix **$\tilde{Z}$** of Z, $\tilde{X}=\tilde{Z}V$ must hold. Let $\tilde{X}$ be the submatrix of X consisting of the first three rows, so $\tilde{X}=2I_{3}$. Accordingly, $\tilde{Z}=\left(\begin{array}{ccc} 2 & 2 & 4\\ 2 & 0 & 0\\ 0 & 2 & 0 \end{array}\right)$. For **$\tilde{X}=\tilde{Z}V$** to be true, the only choice for V is that **$V={\tilde{Z}}^{-1}\tilde{X}=\left(\begin{array}{ccc} 0 & 1 & 0\\ 0 & 0 & 1\\ \frac{1}{2} & -\frac{1}{2} & -\frac{1}{2} \end{array}\right)$**. Nevertheless,

$$\mathrm{ZV}=\left(\begin{array}{ccc} 2 & 2 & 4\\ 2 & 0 & 0\\ 0 & 2 & 0\\ 2 & 1 & 2\\ 1 & 0 & 0\\ 1 & 1 & 1 \end{array}\right)\left(\begin{array}{ccc} 0 & 1 & 0\\ 0 & 0 & 1\\ \frac{1}{2} & -\frac{1}{2} & -\frac{1}{2} \end{array}\right)=\left(\begin{array}{ccc} 2 & 0 & 0\\ 0 & 2 & 0\\ 0 & 0 & 2\\ 1 & 1 & 0\\ 0 & 1 & 0\\ \frac{1}{2} & \frac{1}{2} & \frac{1}{2} \end{array}\right)\neq X,$$

which is a contradiction. In fact, we can clearly see that only the last row of ZV differs from X. So the problem occurs when at least two loci are heterozygous for some genotypes.

## Materials and Methods

### Simulation study

Based on the genomic data of a panel of maize lines belonging to the flint heterotic pool (Bauer *et al.* 2013), simulated traits were generated. Six scenarios were considered with different types and patterns of epistatic QTL effects (Table 3).

**Table 3:** Summary of the six simulation scenarios

**Scenario**	**Additive**	**Epistasis**	**Type of epistasis**	**Pattern of effects**
**1**	Yes	None	None	Independent
**2**	Yes	Global	Digenic	Independent
**3**	Yes	Local	Digenic	Independent
**4**	Yes	Local	Digenic and higher-order	Independent
**5**	Yes	Local	Digenic	Correlated
**6**	Yes	Local	Digenic and higher-order	Correlated

In all scenarios, 100 markers were randomly sampled as QTL for each of the 10 chromosomes, resulting in 1,000 QTL per scenario. In scenario 1, only additive effects were simulated. Hence, the genetic values are g=Ma, where a is the vector of additive QTL effects and M is the marker design matrix. The additive effects were independently sampled from a normal distribution of mean 0 and variance $\sigma_{a}^{2}$, *i.e.*, $a∼N(0,I\sigma_{a}^{2})$. In scenario 2, we simulated additive and global epistatic effects. 1,000 pairs of markers were randomly selected to present digenic epistatic effects. Hence, the genetic values are g=Ma+Faa, where a and **M** are the same as in scenario 1, and aa is the vector of epistatic effects with design matrix **F**. The epistatic effects were also independently sampled from a normal distribution, *i.e.*, **$\mathrm{aa}∼N(0,I\sigma_{aa}^{2})$**.

In scenarios 3 to 6, we simulated additive effects and local epistatic effects. For local epistasis, we first randomly divided each chromosome into non-overlapping blocks, each consisting of 2 to 5 markers. Epistatic effects were simulated only inside individual blocks. Thus, the simulated genetic values $g=\overset{w}{\underset{k=1}{\sum }}Z_{k}\alpha_{k}$, where w is the number of blocks, $\alpha_{k}$ is the vector of additive and epistatic effects inside the k-th block and $Z_{k}$ is the corresponding design matrix. In scenarios 3 and 5, only digenic epistatic effects were simulated. In scenarios 4 and 6, all possible epistatic effects were considered, hence, including higher-order epistasis. In scenarios 3 and 4, all effects were assumed to be independent. In scenarios 5 and 6, epistatic effects inside individual blocks were assumed to be correlated.

For each scenario, we considered one trait with two different simulated heritabilities ($h^{2}$ = 0.7 or 0.5) and two ratios of variances ($\sigma_{a}^{2}/\sigma_{aa}^{2}$ = 4:3 or 3:1). In case of $\sigma_{a}^{2}/\sigma_{aa}^{2}$ = 4:3, the covariance matrices of genetic effects in the individual haplotype blocks were directly derived using the method described in the Theory section, *i.e.*, the covariance matrix equals the matrix VV′, which gave the ratio 4:3 and determined the variances for higher-order epistatic effects. To simulate a situation in which the variance of epistatic effects was less relevant, we considered also a 3:1 ratio. In this case, we modified the matrix VV′ as follows; we changed $\sigma_{aa}^{2}$ from $3\sigma_{a}^{2}/4$ to $\sigma_{a}^{2}/3$ and accordingly modified all variance terms of higher-order epistasis by keeping the ratio of any two epistatic variance terms (*e.g.*, $\sigma_{aa}^{2}/\sigma_{aaa}^{2}$). The variance of additive effects $\sigma_{a}^{2}$ and all correlations were not changed. When only digenic epistatic effects were simulated, the rows and columns corresponding to higher-order epistasis were deleted.

As the final step, the phenotypic values were simulated as y=g+e, where g is the simulated genetic value as described above and e is the environmental error term. The error terms were independently sampled from a normal distribution, *i.e.*, $e∼\;N(0,I\sigma_{e}^{2})$, where $\sigma_{e}^{2}=\frac{1-h^{2}}{h^{2}}\sigma_{g}^{2}$ and $\sigma_{g}^{2}$ is the genetic variance calculated from the simulated genetic values. For each scenario, trait heritability and variance ratio, simulations were repeated 20 times.

### Empirical data

#### Mouse data:

The mouse data set used for this study comprised 1,940 heterogeneous stock mice genotyped with 12,545 SNP markers. The measured traits were body weight at age of six weeks and growth slope between six and ten weeks of age (Valdar *et al.* 2006).

#### Rice data:

The rice data set comprised a diversity panel of 413 varieties genotyped with an Affymetrix 44K SNP array (Zhao *et al.* 2011). Individuals were highly homozygous. After quality control, 39,601 SNP markers were used in this study. Phenotypic data of 26 traits with contrasting genetic architectures were available.

#### Maize data:

The maize data set comprised a large half-sib maize panel from the flint heterotic pool generated within the European PLANT-KBBE CornFed project (Bauer *et al.* 2013). The panel consisted of 11 half-sib families with 833 doubled haploid (DH) lines. After quality control for missing rate and minor allele frequency, 29,466 SNP markers were used for subsequent analyses. Phenotypic traits under consideration were dry matter yield, dry matter content, plant height, days to tasseling and days to silking (Lehermeier *et al.* 2014).

### Genome-wide prediction

**Figure 1:**
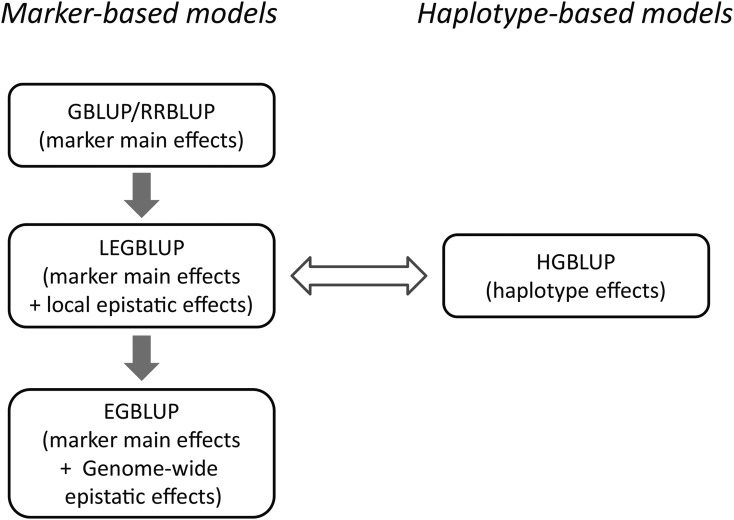
Characteristics and relationships of genomic prediction models considered in this study. The genetic effects exploited by the model were indicated in brackets. GBLUP: genome-wide best linear unbiased prediction; RRBLUP: ridge regression best linear unbiased prediction; EGBLUP: extended genome-wide best linear unbiased prediction; LEGBLUP: locally extended genome-wide best linear unbiased prediction; HGBLUP: haplotype-based genome-wide best linear unbiased prediction. The gray arrows indicate that the models differ with regard to the type and number of effects that are exploited. The equivalence of the LEGBLUP and HGBLUP models that was shown for inbred populations is illustrated by the double arrow.

For the simulated and empirical data, we considered three marker-based models, GBLUP, EGBLUP and LEGBLUP, and one haplotype-based model, HGBLUP (Figure 1). For LEGBLUP and HGBLUP, we defined haplotype blocks using fixed lengths, varying from 2 to 5 (10) SNPs for the simulated (empirical) data. For the mouse data set, in which the linkage phase of the marker data are unknown, we treated each allele of a heterozygous locus as having equal probability (*i.e.*, 50%) to be maternal or paternal. The prediction accuracy (ability) was defined as the Pearson correlation between the predicted and the simulated (observed) genetic values for simulated (empirical) data. For each model the mean prediction accuracy was estimated with fivefold cross validation. All models were implemented using the statistical software R (R Core Team 2016) with the package BGLR (Peréz and de los Campos 2014).

### Data Availability

All empirical data used in this study have been published. The mouse data set was included in the R package SynbreedData (Wimmer *et al.* 2015, https://cran.r-project.org/web/packages/synbreedData/index.html). The rice data set was published in Zhao *et al.* (2011) and can be downloaded from https://ricediversity.org/data/sets/44kgwas/. The genomic data of the maize data set was published in Bauer *et al.* (2013) and can be downloaded from http://www.ncbi.nlm.nih.gov/geo/query/acc.cgi?acc=GSE50558. The phenotypic data of the maize data set was published as File S1 in Lehermeier *et al.* (2014) and can be downloaded from http://www.genetics.org/content/198/1/3.supplemental. Figure S1 shows the prediction accuracies of GBLUP, EGBLUP, LEGBLUP and HGBLUP for simulated traits with heritability 0.5 and $\sigma_{a}^{2}/\sigma_{aa}^{2}$ = 4:3. Figure S2 shows the prediction accuracies of the four models for simulated traits with heritability 0.7 and $\sigma_{a}^{2}/\sigma_{aa}^{2}$ = 3:1. Figure S3 shows the prediction accuracies of the four models for simulated traits with heritability 0.5 and $\sigma_{a}^{2}/\sigma_{aa}^{2}$ = 3:1. Table S1 provides the prediction accuracies of the four models for the 26 agronomic traits in the rice data set. File S1 contains the R code used to generate the data for the simulation study. File S2 and File S3 contain sample genomic and physical map data sets for running the code.

## Results and Discussion

### Modeling haplotype effects exploit local epistasis among markers

We compared two genome-wide prediction models to study whether local epistatic effects among markers are formally taken into account by modeling haplotype effects. The first model utilizes haplotype effects as predictors and has been used in previous studies (Cuyabano *et al.* 2014, 2015a), here we called it HGBLUP. The HGBLUP model is similar to the well-known GBLUP model (VanRaden 2008) which exploits a marker-derived relationship matrix among genotypes. In HGBLUP the marker-derived relationship matrix is replaced by the haplotype-derived relationship matrix. Note that modeling a haplotype-derived relationship matrix is equivalent to explicitly modeling haplotype effects (Equation 1, 2), just like the equivalence between GBLUP and RRBLUP (Habier *et al.* 2007). The second model we considered takes into account additive effects as well as additive-by-additive local epistatic effects among markers and was termed LEGBLUP. LEGBLUP is a modified version of EGBLUP (Jiang and Reif 2015). EGBLUP exploits epistasis between any pair of markers while LEGBLUP only considers local epistasis inside each haplotype block (Equation 3, 4). Note that local higher-order epistatic effects can either be included (Equation 5) or excluded (Equation 3, 4) in the LEGBLUP model. The relationship between the different models was illustrated in Figure 1.

**Figure 2:**
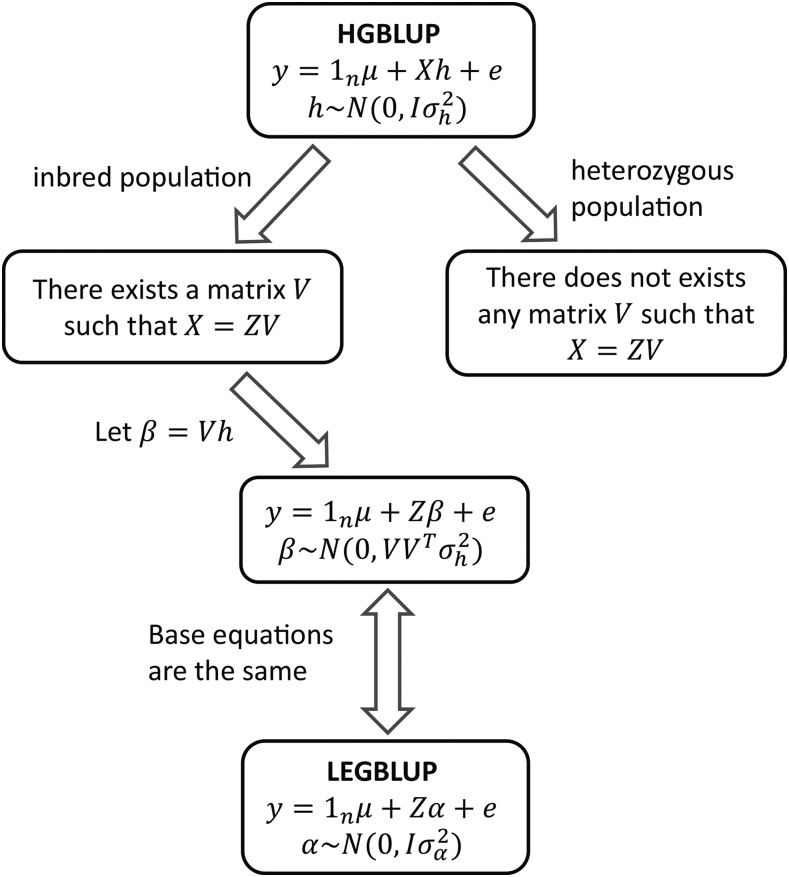
A brief outline of the theoretical relationship between HGBLUP and LEGBLUP. The essential case of a single haplotype block is outlined. LEGBLUP: locally extended genome-wide best linear unbiased prediction; HGBLUP: haplotype-based genome-wide best linear unbiased prediction. In the HGBLUP model, y denotes the vector of observed phenotypic values, $1_{n}$ is the n-dimensional vector of ones where n is the number of genotypes, μ is the common intercept term, h is the vector of haplotype allele effects inside the haplotype block, X is the corresponding design matrix, and e is the residual term. In the LEGBLUP model, α is the vector of main additive and local epistatic effects of all markers inside the haplotype block, Z is the corresponding design matrix, other terms are the same as in HGBLUP. In both models, μ is assumed to be a fixed unknown parameter, h and α are random vectors with distributions shown in the figure, and the residual term $e∼N(0,I\sigma_{e}^{2})$.

A theoretical link between HGBLUP and the full LEGBLUP including local higher-order epistatic effects was established for the case in which all marker loci were assumed to be homozygous. Then the HGBLUP model was proven to be almost statistically equivalent to the LEGBLUP model (Figure 2, and see **Theory** for details). More precisely, the base equation of HGBLUP (Equation 6) is linearly transformable to the one of LEGBLUP (Equation 7). After transformation, only one difference remains; the HGBLUP model assumes non-trivial covariance structure for the additive and local epistatic effects (Equation 8), while in the LEGBLUP model all effects are assumed to be independent (Equation 7). This theoretical derivation provided a formal explanation why and how haplotype-based genome-wide prediction models exploit local epistatic effects among markers.

Note that although almost all genome-wide prediction models assume independent marker effects, it was anticipated that some of the effects may be spatially correlated within chromosomes (Gianola *et al.* 2003). Moreover, it was reported that the prediction accuracy can be increased by the Bayesian antedependence model considering correlated marker effects (Yang and Tempelman 2012). Hence, the covariance structure among the additive and local epistatic effects suggested by the HGBLUP model can be beneficial and is interesting for further study.

A counterexample showed that the base equation of HGBLUP cannot be linearly transformed into the one of LEGBLUP in case that heterozygous loci need to be considered (Figure 2, Example 2 in **Theory**). Hence, further empirical studies are needed to compare HGBLUP with marker-based models to provide more insight into the similarities between marker- and haplotype-based prediction approaches for non-inbred populations.

Our theoretical derivations did not rely on a specific definition of the haplotype blocks in the HGBLUP model. This is important to note since the performance of haplotype-based models has been shown to depend on the method to define the haplotype blocks in experimental studies (Calus *et al.* 2008, 2009, Cuyabano *et al.* 2014, Jónás *et al.* 2016).

### Simulation studies showed that haplotype-based models indeed capture local epistatic effects

**Figure 3:**
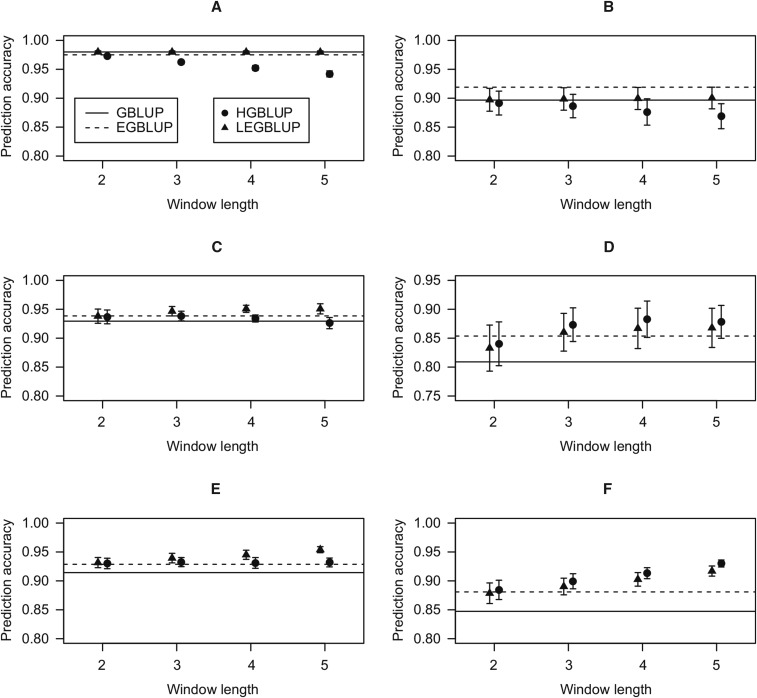
Prediction accuracies of GBLUP, EGBLUP, LEGBLUP, and HGBLUP using simulated data. The data were simulated assuming a trait with the following features; $h^{2}$ = 0.7, $\sigma_{a}^{2}/\sigma_{aa}^{2}$ = 4:3. (a). Scenario 1: only additive effects were simulated; (b) Scenario 2: additive and global epistatic effects were simulated; (c) Scenario 3: additive and digenic local epistatic effects were simulated, effects were assumed to be independent; (d) Scenario 4: additive, digenic and higher-order local epistatic effects were simulated, effects were assumed to be independent; (e) Scenario 5: additive and digenic local epistatic effects were simulated, effects were assumed to be correlated; (f) Scenario 6: additive, digenic and higher-order local epistatic effects were simulated, effects were assumed to be correlated; GBLUP: genome-wide best linear unbiased prediction; EGBLUP: extended genome-wide best linear unbiased prediction; LEGBLUP: locally extended genome-wide best linear unbiased prediction; HGBLUP: haplotype-based genome-wide best linear unbiased prediction. Standard errors of the estimated prediction accuracies are indicated by whiskers. The LEGBLUP and HGBLUP models were implemented with different window length (*i.e.*, number of SNPs), varying from 2 to 5.

Simulation studies were used to scrutinize that the HGBLUP model exploits local epistatic effects among markers. Six scenarios which differed with respect to the nature and pattern of epistatic effects were utilized (Table 3) to compare the performance of HGBLUP with those of GBLUP and EGBLUP (Figure 3). In scenarios in which no local epistatic effects were simulated, the highest prediction accuracies were achieved by GBLUP (Figure 3a) and EGBLUP (Figure 3b) and no benefit was observed for HGBLUP. In the four scenarios in which local epistasis was simulated, considering window sizes from 3 to 5 HGBLUP clearly outperformed GBLUP and EGBLUP in two cases (Figure 3d, f), but not in the scenarios in which only digenic local epistatic effects were simulated (Figure 3c, e). According to the theoretical derivation, the HGBLUP model assumes correlated local epistatic effects and considers not only local digenic but also higher-order epistatic effects among markers. This explains why the HGBLUP model did not perform well in scenarios in which the latter assumption was not fulfilled.

The results shown in Figure 3 were obtained for a trait with a simulated heritability of 0.7 and a ratio of 4:3 for the simulated variance of additive effects to that of epistatic effects, $\sigma_{a}^{2}/\sigma_{aa}^{2}$. The ratio 4:3 represents an optimized ratio for HGBLUP as it was derived in the linear transformation from HGBLUP to LEGBLUP (see **Materials and Methods** for details). We observed that the findings in case of a lower heritability of 0.5 in conjunction with $\sigma_{a}^{2}/\sigma_{aa}^{2}$ equaling 4:3 followed the same pattern (Figure S1), suggesting that the conclusions are valid for traits with a range of heritabilities. If $\sigma_{a}^{2}/\sigma_{aa}^{2}$ was set to 3:1, the advantage of HGBLUP was reduced in scenarios in which higher-order local epistatic effects were simulated (Figure S2d, f and Figure S3d, f). These results are expected as the relevance of epistasis was purposely weakened by the applied ratio of 3:1 for $\sigma_{a}^{2}/\sigma_{aa}^{2}$. In summary, the results of the simulation studies confirm that local epistasis is indeed exploited by the HGBLUP model.

### Haplotype-based models are especially useful when local higher-order epistasis is important

Our theoretical derivations showed that the haplotype-based model HGBLUP is able to exploit local epistatic effects among markers, since HGBLUP and the marker-based model LEGBLUP were shown to be almost statistically equivalent. As a next step, we asked under which circumstances the haplotype-based model outperforms the marker-based model. In order to minimize the demand on computational resources, discussed in detail in the next subsection, we implemented the LEGBLUP model such that only additive and digenic local epistatic effects were considered (Equation 3). Under these constraints, two differences exist between HGBLUP and LEGBLUP. First, higher-order local epistasis is considered in HGBLUP but not in LEGBLUP. Second, HGBLUP assumes correlated local epistatic effects, while LEGBLUP assumes independent effects. The relative impact of these factors was assessed by comparing the performances of HGBLUP and LEGBLUP in our simulation study. In scenarios in which higher-order local epistasis was simulated, HGBLUP outperformed LEGBLUP regardless whether correlated or independent local epistatic effects were simulated (Figure 3d, f). In contrast, in scenarios in which only digenic local epistasis was simulated, the prediction accuracies of LEGBLUP were higher than those of HGBLUP (Figure 3c, e). In scenario 4 (Figure 3d), the assumption that local epistatic effects were independent should have favored LEGBLUP, nonetheless HGBLUP outperformed LEGBLUP suggesting that the influence of the effect pattern was masked by the inclusion of higher-order local epistasis. In scenario 5 (Figure 3e), local epistatic effects were assumed to be correlated, this should have favored HGBLUP, yet LEGBLUP yielded higher prediction accuracies than HGBLUP, indicating that the exclusion of higher-order epistasis had a stronger effect than the effect pattern. Thus, among the assumptions favoring HGBLUP, the presence of higher-order local epistasis was found to be the most important. This conclusion holds for a range of simulated heritabilities (Figure S1). However, when the ratio of the simulated variance of additive effects to that of epistatic effects $\sigma_{a}^{2}/\sigma_{aa}^{2}$ increased the advantage of HGBLUP decreased (Figure S2d, f) and/or even disappeared at certain window sizes (Figure S3d, f).

The contribution of higher-order epistasis to the phenotypic variation of complex traits is poorly understood because higher-order epistasis is difficult to detect in genetic mapping studies (Taylor and Ehrenreich 2015). Nevertheless, evidences for higher-order gene interactions from model organisms were reported (Pettersson *et al.* 2011, Taylor and Ehrenreich 2014) and new approaches were developed to detect them (Sailer and Harms 2017). The comparisons of the prediction accuracies of HGBLUP *vs.* single marker-based approaches pave the way for a new approach to provide insights into the relevance of higher-order epistasis for complex traits.

### Haplotype-based models are computationally efficient in exploiting local epistasis

In the analyses of the experimental data, the LEGBLUP model was implemented in a way that only additive and digenic epistatic effects were included (Equation 3). Thus, two kinship matrices were considered in the LEGBLUP model, the additive kinship matrix and the digenic local epistatic kinship matrix. In contrast, the HGBLUP model is based on a single kinship matrix. We compared the speed of HGBLUP and LEGBLUP with 100 cross validations using a maize data set with 833 individuals and 29,466 SNP markers (see **Materials and Methods**). The computer used for the test was equipped with Intel(R) Core(TM) i7-6700 CPU (3.40 GHz) and 32.0 GB RAM. The computational time was with 51 min for the LEGBLUP model nearly twice as long compared to the HGBLUP model which took only 28 min. Although the full LEGBLUP model potentially may yield comparable prediction accuracies as HGBLUP when higher-order epistasis is relevant, it would be far less efficient than HGBLUP, therefore we did not implement the full LEGBLUP model which includes local higher-order epistasis in our data analyses. In summary, the haplotype-based model HGBLUP is computationally much more efficient in exploiting local epistasis compared to marker-based models. This point may be of particular relevance for future studies since ultra-high density SNP data sets are emerging for plant and animal populations owing to the rapid progress with regard to genotyping-by-sequencing approaches (Scheben *et al.* 2017).

### The performance of haplotype-based genome-wide prediction models in empirical data sets

Our theoretical and simulation results have shown that the HGBLUP model increases the prediction accuracy for inbred populations when local epistasis is abundant. To explore the potential of HGBLUP, we compared the performance of HGBLUP with the three marker-based models GBLUP, EGBLUP, and LEGBLUP using one animal data set and two crop data sets.

**Figure 4:**
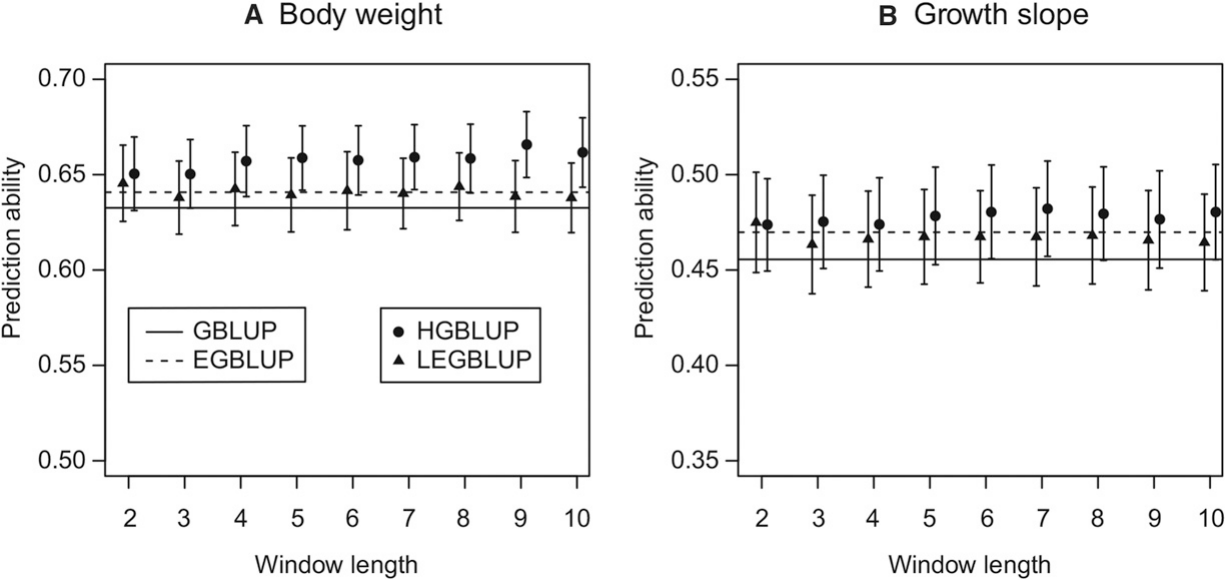
Prediction abilities of GBLUP, EGBLUP, LEGBLUP and HGBLUP for the mouse data set. GBLUP: genomic best linear unbiased prediction; EGBLUP: extended genomic best linear unbiased prediction; LEGBLUP: locally extended genomic best linear unbiased prediction; HGBLUP: haplotype-based genomic best linear unbiased prediction. Standard errors of the estimated prediction abilities are indicated by whiskers. The LEGBLUP and HGBLUP models were implemented with different window length (*i.e.*, number of SNPs), varying from 2 to 10.

The mouse data set comprised non-inbred genotypes. For both analyzed traits (Figure 4), the HGBLUP model clearly outperformed the other three models, suggesting that the HGBLUP model also exploits local epistasis in case heterozygous loci need to be considered. This result is of particular relevance since it was not possible to prove theoretically that the HGBLUP model is able to exploit local epistatic effects in case of non-inbred populations (Figure 2). Given that haplotype-based genome-wide prediction models have been successfully applied in non-inbred cattle populations and outperformed alternative marker-based models (Boichard *et al.* 2012, Cuyabano *et al.* 2014, 2015a, b, Jónás *et al.* 2016), the haplotype-based genome-wide prediction model is an attractive tool for non-inbred populations.

**Figure 5:**
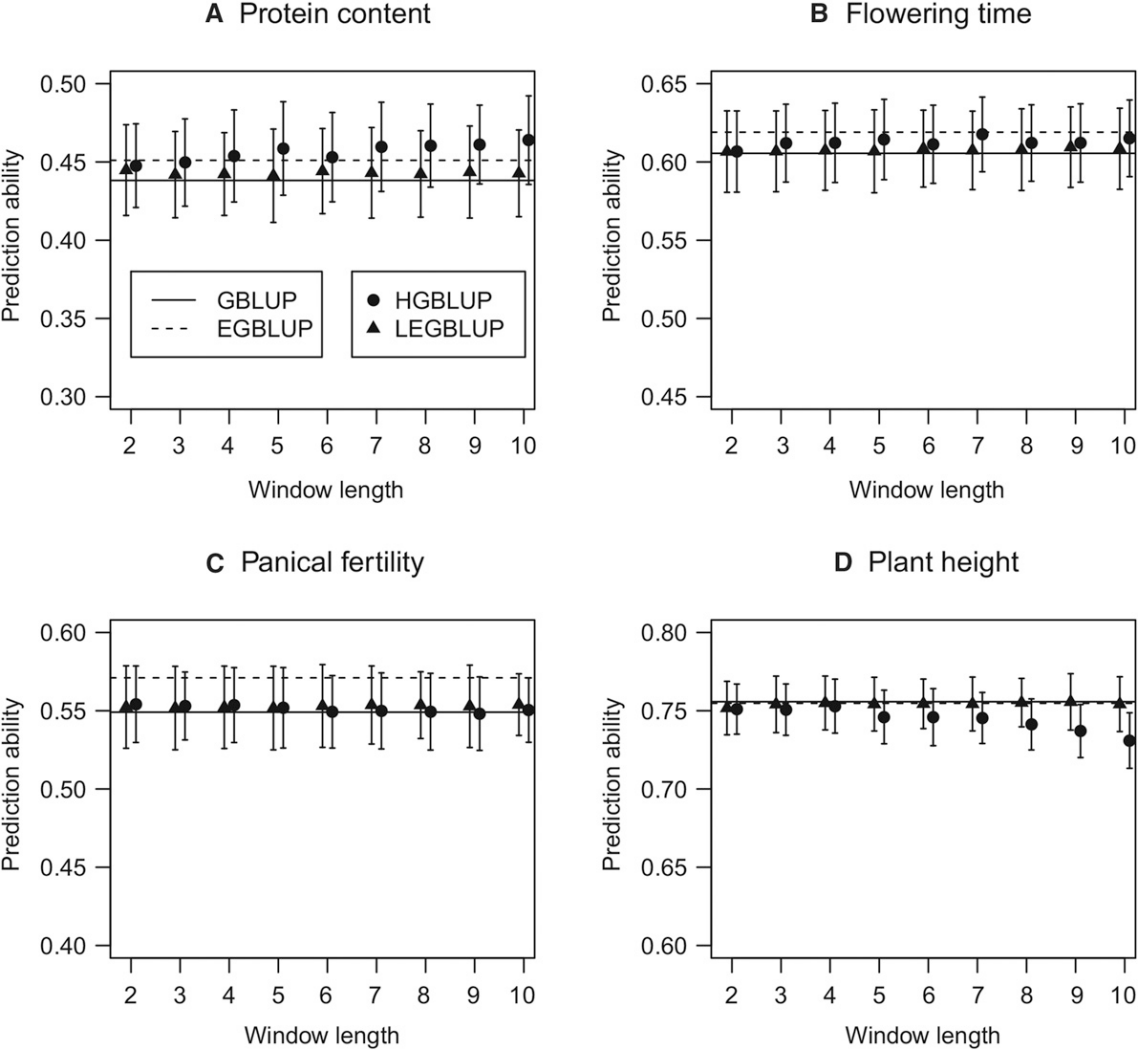
Prediction abilities of GBLUP, EGBLUP, LEGBLUP and HGBLUP for the rice data set. GBLUP: genomic best linear unbiased prediction; EGBLUP: extended genomic best linear unbiased prediction; LEGBLUP: locally extended genomic best linear unbiased prediction; HGBLUP: haplotype-based genomic best linear unbiased prediction. Whiskers indicate standard errors of the estimated prediction abilities. The LEGBLUP and HGBLUP models were implemented with different window length (*i.e.*, number of SNPs), varying from 2 to 10.

For the rice data set, 26 agronomic traits (Zhao *et al.* 2011) with different genetic architectures were evaluated (Table S1). We observed that for three traits, such as protein content, HGBLUP outperformed all other models (Figure 5a). For two traits, including flowering time, HGBLUP gave slightly higher prediction accuracies than GBLUP and LEGBLUP, but lower ones than EGBLUP (Figure 5b). There were six traits for which only EGBLUP outperformed the other models, as shown for panicle fertility (Figure 5c). For the remaining fifteen traits, including plant height, GBLUP yielded the best prediction accuracies (Figure 5d).

**Figure 6:**
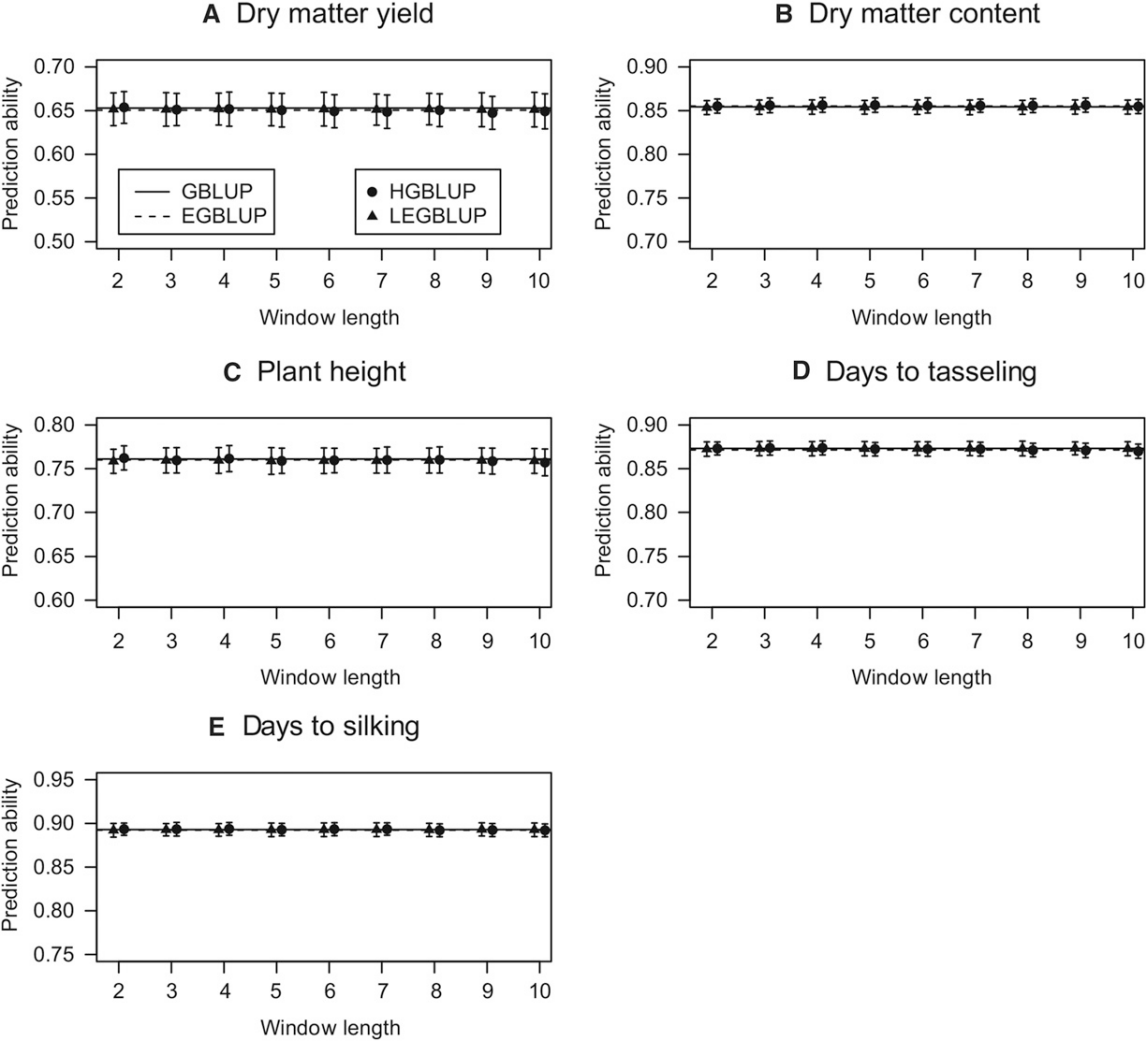
Prediction abilities of GBLUP, EGBLUP, LEGBLUP and HGBLUP for the maize data set. GBLUP: genomic best linear unbiased prediction; EGBLUP: extended genomic best linear unbiased prediction; LEGBLUP: locally extended genomic best linear unbiased prediction; HGBLUP: haplotype-based genomic best linear unbiased prediction. Standard errors of the estimated prediction abilities are indicated by whiskers. The LEGBLUP and HGBLUP models were implemented with different window length (*i.e.*, number of SNPs), varying from 2 to 10.

For the maize data set, HGBLUP provided no benefit for the five traits under consideration. In fact, in all cases the best prediction accuracy was observed for the GBLUP model which only takes additive effects into account (Figure 6).

The contrasting results we observed for different traits in the crop data sets indicated that the haplotype-based model will not generally boost prediction accuracies in crop populations. Instead, the effectiveness of HGBLUP may depend on the complexity of the trait. Analysis of the trait flowering time in rice and maize revealed that HGBLUP increased prediction accuracies in rice (Figure 5b), but not in maize (Figure 6d, e). As a matter of fact, HGBLUP failed to increase prediction accuracies regardless which trait was analyzed for the maize data set, in contrast to the results for the rice data set. These findings are in accordance with those obtained in a recent study (Akdemir and Jannink 2015) where a semiparametric mixed model with multiple marker-derived local epistatic genomic relationship matrices was applied to wheat, barley, and maize data. It was observed that the local epistatic model performed well in the wheat and barley data sets but not in the maize data set, possibly indicating the different relevance of epistasis in selfing and outcrossing species (Garcia *et al.* 2008).

As the models GBLUP, EGBLUP and HGBLUP capitalize on different genetic effects in prediction, comparing the prediction accuracies of these models provides a first insight into the genetic architecture of a particular trait in a given organism. There is however a risk of misinterpreting local epistatic effects due to “apparent epistasis” (Wood *et al.* 2014), a phenomenon which refers to the fact that multi-locus genotype tags may mimic tight linkage disequilibrium with an unobserved functional variant in the genome for a single marker. In such a case, the HGBLUP model would actually exploit the hidden additive effects of the unobserved variants, instead of the local epistatic effects. The fact that HGBLUP incorporates both additive and local epistatic effects for prediction is of particular relevance for breeders; in cases in which HGBLUP outperforms GBLUP, local epistatic effects or effects that are due to apparent epistasis are expected to be passed on for several generations, very much like additive effects.

## Conclusions

In this study, we investigated the relationship between haplotype-based and marker-based genome-wide prediction models. We provided a mathematical proof that modeling haplotype effects is equivalent to modeling main and local epistatic effects of markers, but with a different covariance matrix. Our simulation study confirmed the theoretical results and revealed that haplotype-based models are superior to marker-based models when there is abundant higher-order local epistasis. The fact that haplotype-based models exploit local epistasis among markers is especially relevant for applied breeding as the local additive-by-additive epistatic effects can last for generations like the additive effects. Thus, haplotype-based models have the potential to increase the accuracy of genomic selection. This hypothesis was partly supported by our empirical data analyses as we observed in certain cases that modeling local epistasis is indeed better than only modeling main effects. Further studies are needed to find out for which traits and in which species the haplotype-based models can be beneficial in genomic selection.
